# *Laccase-13* Regulates Seed Setting Rate by Affecting Hydrogen Peroxide Dynamics and Mitochondrial Integrity in Rice

**DOI:** 10.3389/fpls.2017.01324

**Published:** 2017-07-26

**Authors:** Yang Yu, Quan-Feng Li, Jin-Ping Zhang, Fan Zhang, Yan-Fei Zhou, Yan-Zhao Feng, Yue-Qin Chen, Yu-Chan Zhang

**Affiliations:** State Key Laboratory for Biocontrol, School of Life Science, Sun Yat-sen University Guangzhou, China

**Keywords:** rice, seed setting rate, laccase, hydrogen peroxide, mitochondria

## Abstract

Seed setting rate is one of the most important components of rice grain yield. To date, only several genes regulating setting rate have been identified in plant. In this study, we showed that *laccase-13* (*OsLAC13*), a member of laccase family genes which are known for their roles in modulating phenylpropanoid pathway and secondary lignification in cell wall, exerts a regulatory function in rice seed setting rate. *OsLAC13* expressed in anthers and promotes hydrogen peroxide production both *in vitro* and in the filaments and anther connectives. Knock-out of *OsLAC13* showed significantly increased seed setting rate, while overexpression of this gene exhibited induced mitochondrial damage and suppressed sugar transportation in anthers, which in turn affected seed setting rate. *OsLAC13* also induced H_2_O_2_ production and mitochondrial damage in the root tip cells which caused the lethal phenotype. We also showed that high abundant of OsmiR397, the suppressor of *OsLAC13* mRNA, increased the seed setting rate of rice plants, and restrains H_2_O_2_ accumulation in roots during oxidative stress. Our results suggested a novel regulatory role of *OsLAC13* gene in regulating seed setting rate by affecting H_2_O_2_ dynamics and mitochondrial integrity in rice.

## Introduction

Rice is one of the most important food crops. Grain size, grain number, and panicle number are the determinants of rice grain yield. Seed setting rate determines grain number and is susceptive to environmental conditions, which often lead to decrease of rice yield. Several genes related to seed setting rate have been reported in rice, such as *PTB1* positively regulate seed setting rate by controlling pollen tube growth (Li et al., 2013), *GSD1* affects seed setting rate through regulating plasmodesmatal conductance (Gui et al., 2014), and *THIS1* regulates both seed setting and plant architecture (Liu et al., 2013). In higher plants, male reproductive organogenesis requires the establishment of anthers and filaments. Abnormal reproductive organogenesis also reduces seed setting rate. For example, *OsSPX1*, a rice SPX domain gene, is involved in anther and pollen development. Down-regulation of *OsSPX1* leads to semi-male sterility and ultimately resulted in low seed-setting rate and grain yield (Zhang et al., 2016). Knock-out of *GSL5* which encodes the callose synthase disrupts normal microspore development during late meiosis and exhibits a severe reduction of seed setting rate (Shi et al., 2015). At the late stage of pollen maturation, starch accumulates in the pollen as an energy reserve for germination. Thus, starch accumulation serves as a marker of pollen maturity (Datta et al., 2002). As a non-photosynthetic organ, the anther obtains sugars mainly from source organs such as leaves and sink organs such as lemma and palea (Goetz et al., 2001). The connective attaches the anther to the filament, which acts as the conduit and provides a link for vascular transport of photosynthetic sugars and other essential nutrients to the anther and the sugars deposited as starch in the pollen provide energy for development following pollination (Cardarelli and Cecchetti, 2014). However, the importance of filaments in anther development and male fertility has not been studied in detail.

Plants generate reactive oxygen species (ROS) by using molecular oxygen as a terminal electron acceptor, creating molecules such as superoxide anion (O_2_^-^), hydroxyl radicals (OH^-^), and hydrogen peroxide (H_2_O_2_) (Hu et al., 2011). ROS are highly reactive and toxic in damaging proteins, lipids, DNA, and carbohydrates (Gill and Tuteja, 2010). Moreover, recent work has identified ROS, particularly H_2_O_2_, as important second messengers in signal transduction networks that regulate plant developmental processes such as cell expansion, polar growth, flower development, and stress responses (Alvarez et al., 1998; Skopelitis et al., 2006). Notably, several recent studies showed that ROS affect pollen maturation and male fertility by accumulating in the tapetum and pollen tube (Wu et al., 2010; Hu et al., 2011; Xie et al., 2014), suggesting that ROS serve as important regulatory molecules for male reproductive development.

As one of the evolutionarily oldest enzymes in both fungi and plants, laccases (LACs) have been studied for years. Most studies on plant laccases have mainly focused on secondary lignification in cell walls (Berthet et al., 2011; Lu et al., 2013; Zhao et al., 2013; Wang et al., 2014; Bryan et al., 2016), via the phenylpropanoid pathway (Vogt, 2010). However, LACs have a wide range of substrates and thus might have diverse and complicated functions. Indeed, recent studies showed that LACs in higher plants have more varied functions than expected. For example, our recent study showed that LACs regulate grain yield in both rice and *Arabidopsis thaliana* (Zhang et al., 2013; Wang et al., 2014). Other *LAC* genes also affect seed coat color and nutrient transportation in *Arabidopsis* (Turlapati et al., 2011), suggesting that LACs affect important plant traits.

In this study, we reported a novel function of *OsLAC13* in regulating seed setting rate and H_2_O_2_ dynamics in rice. Knock-out of *OsLAC13* increases seed setting rate. By contrast, higher expression level of *OsLAC13* reduces seed setting rate dramatically by inducing H_2_O_2_ accumulation in filaments and anther connectives and suppressing the maturation of pollen grains. The integrity of mitochondria was damaged both in the phloem cells of vascular tissue and in the root tip cells when elevating the expression level of *OsLAC13*. We also showed that *OsLAC13* is under the regulation of OsmiR397 during anther development and stress response of rice plants. Our data therefore report a novel regulatory role of *OsLAC13* gene in regulating H_2_O_2_ dynamics and seed setting rate in rice.

## Materials and Methods

### Plant Growth Conditions and Generation of Transgenic Rice Plants

The growth conditions and generation of transgenic plants were conducted as described by Zhang et al. (2013). Briefly, the Zhonghua 11 (*Oryza sativa japonica*) rice cultivar was used in the experiments. Rice plants were grown in the field in Guangzhou, China (23°08′ N, 113°18′ E), where the growing season extends from late April to late September. The average low temperature range is ∼22.9–25.5°C, and the average high temperature range is ∼29.7–32.9°C. The day length ranged from 12 to 13.5 h. Plants were maintained with routine management practices. *OsLAC13*, pre-OsmiR397a, pre-OsmiR397b, and pre-mmiR397 (pre-mmiR397 contains several mismatches to the OsLAC binding site but can also produce a 21-nt small RNA) were overexpressed under the control of the CaMV35S promoter. The *OsLAC13*-RNAi transgenic plants were generated using pRNAi-35S vectors, which cloned the *OsLAC13* gene fragments in the sense and antisense orientations, and the construct expressing the RNA hairpin was driven by the CaMV35S promoter. T_3_ seeds that were homozygous for the transgene were harvested and several lines with high expression levels were used for further analysis. The OsLAC13 knock-out muntants were constract by CRISPR-Cas9 based genome editing technology as discribed (Ma et al., 2015). The primers are as follows. Target site 1: 5′-ggcAgcagcaacgaagaacagagg-3′ and 3′-cgtcgttgcttcttgtctcccaaa-5′. Target site 2: 5′-gccGtacgtgtgcgtgcaggcac-3′ and 3′-atgcacacgcacgtccgtgcaaa-5′.

### DAPI Staining

The 4′,6-diamidino-2-phenylindole (DAPI) staining was performed essentially as described previously, with minor modifications (Ross et al., 1996). The fixed tissue was washed twice with water and twice with 10 mM citrate buffer, pH 4.5. Four to six anthers were placed in a small drop of 60% acetic acid on a slide and pressed with another slide to release microspore mother cells. The slides were then separated and the samples dried at room temperature for 5 min. A total of 5 μL DAPI solution (1 μg/mL DAPI in a buffer with 50% glycerol and 10 mM citrate, pH 4.5) was placed onto the slide, which was then covered with a cover glass and sealed with nail polish. Slides were examined under a fluorescence microscope (Leica DM5000B).

### Transmission Electron Microscopy

Samples were fixed in 5% (w/v) glutaraldehyde, 4% paraformaldehyde in 0.1 M sodium phosphate buffer, pH 7.0, and were then post-fixed in 2% OsO_4_ in PBS, pH 7.2. Following ethanol dehydration, samples were embedded in acrylic resin. Ultrathin sections (50–70 nm) were double-stained with 2% (w/v) uranyl acetate and 2.6% (w/v) lead citrate aqueous solution and examined with a JEOL JEM – 100CX II transmission electron microscope.

### *In Vitro* Pollen Germination Assay

Pollen germination tests were performed as described previously (Zhou et al., 2011). Briefly, pollen grains were placed on a clean cover glass, and 20 μL of Brewbaker and Kwach medium (10% sucrose, 100 mg/L boric acid, 300 mg/L calcium nitrate, 200 mg/L magnesium sulfate, and 100 mg/L potassium nitrate) were added. The cover glass was placed in a humid dish, and incubated for 60 min at 25°C in the dark. The pollen grains were then observed under a microscope. Pollen grains with a pollen tube elongated longer than the diameter of the pollen grain were scored as successful germination.

### Soluble Sugar Assays by GC-MS and Measurement of Starch

Metabolites were analyzed essentially as described, with modifications (Lisec et al., 2006). Briefly, about 50 mg (fresh weight) of anther, lemma, palea, or flag leaf was harvested and ground into a fine powder in liquid nitrogen. To stop enzymatic activity, 700 μL methanol was immediately added to the powder and 120 μL of 0.2 mg/mL rabitol (Sigma–Aldrich) was then added. The mixture was shaken at 950 RPM at 70°C for 10 min. After centrifugation at 11,000 g for 10 min, the supernatant was transferred to a new tube and dried for sugar assays; the remaining pellet was used to assay starch contents using a starch assay kit (product number SA20-1KT; Sigma–Aldrich). For sugar assays, 40 μL of methoxyamination reagent was used at 37°C for 2 h. Afterward, 40 μL of *N*-methyl-*N*-(trimethylsilyl) trifluoroacetamide was added, and the mixture was incubated at 37°C for 30 min. GC–MS analysis was performed using an Agilent 6890 series gas chromatograph fitted with a capillary column (0.25 mm × 30 m, 0.25 mm film thickness [HP–5MS]). The gas chromatograph was combined with a quadrupole mass selective detector (Agilent).

### Histochemical Assays of Superoxide Anion and Hydrogen Peroxide

*In vivo* hydrogen peroxide staining was performed as described by Barcelo (1998) using TMB. Freshly collected spikelets were put in the staining solution (0.1 mg/mL TMB in Tris-acetate, pH 5.0) under vacuum conditions for 15 min and then incubated at 25°C until the blue color appeared. Production of superoxide anion was visualized by incubating intact anthers in 10 mM K-citrate buffer, pH 6.0, containing 0.5 mM NBT (Liszkay et al., 2004).

### *In Vitro* Induction of H_2_O_2_ by *OsLAC13*

For mammalian expression system, full-length *OsLAC13* gene was contrasted into pCDH-CMV-MCS-EF1-Puro vectors that inserted eGFP. Then, 15ug *OsLAC13*-pCDH vector were transfected with Lipofectamine LTX (Invitrogen Corporation, Carlsbad, CA, United States) into the 3 × 106 293T cells plated in 10 cm dish before 24 h. Add 60 μl of Premo^TM^ Cellular H_2_O_2_ Sensor (Moleqular probes) per 75,000 cells in growth medium. Finally, 48 h after transfection, cells were analyzed using a confocal laser scanning microscope (Zeiss 7 DUO NLO) at 400- and 488-nm excitation, and emission at 515-nm.

For rice protoplast transient transfection, *OsLAC13* was overexpressed under the control of the CaMV35S promoter. Two-week-old rice shoots were used to isolate protoplast. A bundle of rice plants (approximately 30 seedlings) was cut together into approximately 0.5 mm strips with propulsive force using sharp razors. The strips were incubated in an enzyme solution (1.5% cellulose RS, 0.75% macerozyme R-10, 0.6 M mannitol, 10 mM MES at pH 5.7, 10 mM CaCl2 and 0.1% BSA) for 4–5 h in the dark with gentle shaking (40–50 rpm). After the enzymatic digestion, an equal volume of W5 solution (154 mM NaCl, 125 mM CaCl2, 5 mM KCl, and 2 mM MES at pH 5.7) was added, followed by shaking (60–80 rpm) for 30 min. Protoplasts were released by filtering through 40-μm nylon mesh into round bottom tubes, washing the strips with W5 solution 3–5 times. The pellets were collected by centrifugation at 800 rpm for 3 min in a swinging bucket. After washing once with W5 solution, the pellets were then resuspended in MMG solution (0.4 M mannitol, 15 mM MgCl2 and 4 mM MES at pH 5.7) at a concentration of 2 × 106 cells mL-1. PEG-mediated transfections were carried out as previously described (Zhang et al., 2011). Protoplasts were observed 24 h after transfection using a confocal laser scanning microscope (Zeiss 7 DUO NLO) at 400- and 488-nm excitation, and emission at 515-nm. Each experiment was repeated at least three times.

## Results

### *OsLAC13* Regulates Seed Setting Rate and Male Reproductive Organogenesis in Rice

**FIGURE 1:**
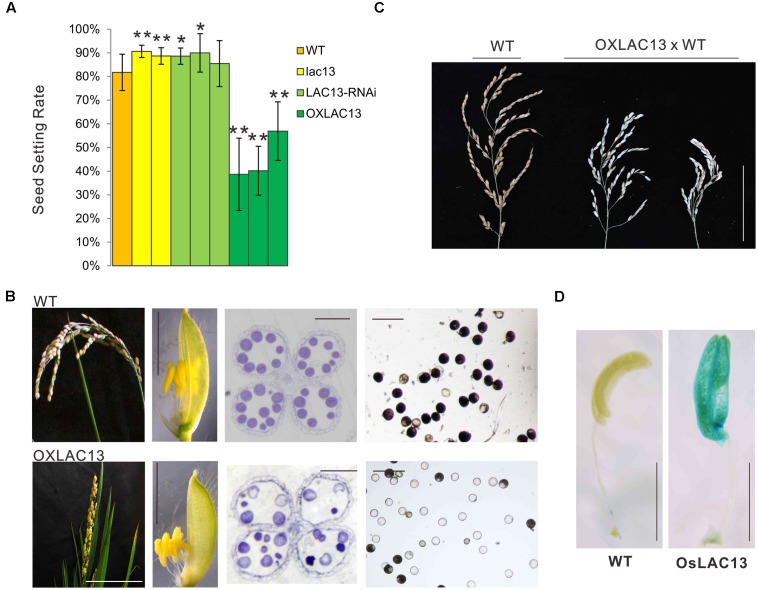
*OsLAC13* regulates seed setting rate and male reproductive organogenesis. **(A)** The seed setting rates of WT, *OsLAC13* knock out (*lac13*), *OsLAC13*-RNAi, and OXLAC13 plants. Values are the means ± s. d. (*n* = 40 plants). Significant differences were identified at the 5% (^∗^) and 1% (^∗∗^) probability levels using Student’s *t*-test. **(B)** The panicles of OXLAC13 plants crossed with WT plants. Scale bars, 10 cm. **(C)** The panicles (scale bars, 2.5 cm), the spikelet (scale bars, 4 mm), the transverse semithin sections of mature anthers (scale bars, 100 μm) and the mature pollen grains stained for starch with I_2_-KI (scale bars, 100 μm) of WT and OXLAC13 plants from the left panels to the right panels. **(D)** Expression patterns of *OsLAC13* by GUS staining (Blue). Scale bars, 2 mm.

To identified the functions of *OsLAC13*, we first constructed the transgenic plants that knockout of *OsLAC13* (*lac13*) by CRISPR-Cas9 based genome editing technology (Supplementary Figure 1A) and analyzed their phenotypes. We found that the seed setting rate (the ratio of number of filled grains to total number of spikelets) of the *lac13* plants increased significantly compared with that of the wild-type (WT) plants. Consistently, the seed setting rate was decreased dramatically in the *OsLAC13* over-expression plants (OXLAC13), and was increased slightly in the *OsLAC13* RNA interference lines (*LAC13* RNAi) (**Figure 1A** and Supplementary Figure 1B). These results indicated *OsLAC13* plays a role in regulating seed setting rate in rice.

We then analyzed the floral developmental process of the transgenic plants to identify how *OsLAC13* control seed setting rate. The phenotypical analysis showed that, compared with wild-type and the *lac13* plants, the OXLAC13 plants showed unfavorable characteristics including small panicles, lethal phenotype and semi-sterility (**Figure 1B**). The OXLAC13 pistils are developed normally (Supplementary Figure 1D). The OXLAC13 plants produced apparently normally developed but slightly smaller spikelets and anthers, although less than 1% of the anthers appeared twisted (**Figure 1B** and Supplementary Figure 1E). The decreased seed setting rate in the OXLAC13 plants was induced by abnormal male reproductive organogenesis, that most of the mature pollen grains lacked starch, as revealed by iodine-potassium iodide staining of semi-thin sections (**Figure 1B**). The hybrids of the OXLAC13 plants and the WT plants also showed semi-sterile phenotype (**Figure 1C**). These results suggested that *OsLAC13* restrains male reproductive organogenesis and negatively regulates rice setting rate.

### *OsLAC13* Restrains Carbohydrate Transportation to Anthers and Filament Elongation

To further characterize the role of *OsLAC13* in anther development, we analyzed the spatial expression patterns of *OsLAC13* in anthers byβ-glucuronidase (GUS) activity analysis. *OsLAC13* is highly expressed in anthers, especially in anther connectives (**Figure 1D**). We then performed a detailed analysis of anther morphology. The OXLAC13 plants undergoes normal meiosis, as revealed by DAPI staining (Supplementary Figure 2A), indicating that high levels of *OsLAC13* transcripts does not affect meiosis. We also investigated the development of the microspores of the OXLAC13 plants after meiosis, and found no obvious phenotypic alterations. The pollen grains developed normally to the trinucleate stage, but had slow starch deposition in the spores, as shown by toluidine blue staining of semi-thin sections (Supplementary Figures 2B,C). These results indicated that the OXLAC13 anthers have no defects during microspore development except for suppressed nutrient accumulation in anthers and pollen grains.

**FIGURE 2:**
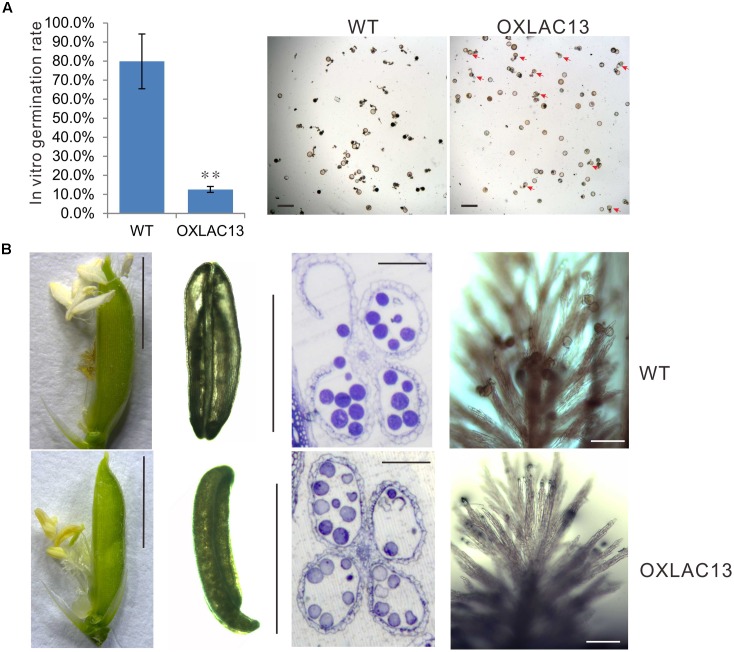
Defects of OXLAC13 plants in late anther development. **(A)** *In vitro* germination rates of WT and OXLAC13 pollen. Scale bars, 100 μm. Values are means ± s. d. (*n* = 200 pollen grains). Red arrows indicate the succesfully germinated OXLAC13 pollen grains. Significant differences were identified at the 5% (^∗^) and 1% (^∗∗^) probability levels using Student’s *t*-test. **(B)** Spikelets 1 day after flowering (Scale bars, 4 mm), anthers after flowering (Scale bars, 2 mm), transverse sections of anthers after flowering (Scale bars, 100 μm) and the stigmas 2 h after flowering (Scale bars, 100 μm) of WT and OXLAC13 plants from the left panels to the right panels.

In the mature anthers, the WT pollen grains were deeply stained, indicating that they contained stored starch, but the OXLAC13 pollen grains were almost unstained, showing the failure of starch deposition (**Figure 1B**). We also monitored the *in vitro* germination rate of pollen grains. The results showed that the WT pollens had a germination rate of ∼79.8%, but the OXLAC13 pollens only had a germination rate of ∼12.6% (**Figure 2A**). This result was generally consistent with the result of iodine-potassium iodide staining. We further analyzed the sugar contents of the carbohydrate source tissues (flag leaf, lemma, and palea) during anther development (Zhang et al., 2010) to examine whether the reduction of starch accumulation in the OXLAC13 pollen grains was caused by failure of carbohydrate synthesis in the OXLAC13 plants. However, the results showed that the source tissues of the OXLAC13 plants had similar or only slightly higher levels of sugars (sucrose, glucose, and fructose) and starch as that of the WT plants at both the stages that before and after starch deposition in pollen grains, implying that the OXLAC13 plants have no defect in carbohydrate synthesis (Supplementary Figures 3A–C). The anther vascular tissues from filaments to anther connectives is responsible for transporting nutrients to anthers, thus the roles of anther vascular tissues might be blocked in the OXLAC13 plants.

Noticeably, we also observed a failure of filament elongation in the OXLAC13 spikelets (**Figure 2B**). At the flowering stage, the WT filaments elongated and the anthers reach the top of the spikelet, and then dehisce, releasing pollen grains over the stigma of the pistil for pollination in the WT plants. After anthesis, the spikelet remains closed and the empty anthers are outside the spikelet (**Figure 2B**). However, the filaments of about 43.9% of the OXLAC13 spikelets failed to elongate and about 40.2% of the anthers did not dehisce (**Figure 2B**). Consistent with this, only 37.3% of the OXLAC13 stigmas had more than 20 pollen grains when over 93% of the WT stigmas had more than 20 pollen grains at 2 h after anthesis, and 42.4% of the OXLAC13 stigmas did not have any pollen grains at all (**Figure 2B**), showing that the semi-sterility of the OXLAC13 plants is caused by a series of defects in late anther development and pollination, including filament elongation. Consider the expression patterns of *OsLAC13*, together with these results, it could be speculated that *OsLAC13* possibly affects the roles of anther vascular tissue including sugar transportation from source tissues to anthers, and filaments elongation.

### *OsLAC13* Induces Hydrogen Peroxide Production and Affects the Number and Integrity of the Mitochondria in Stamen Vascular Cells

**FIGURE 3:**
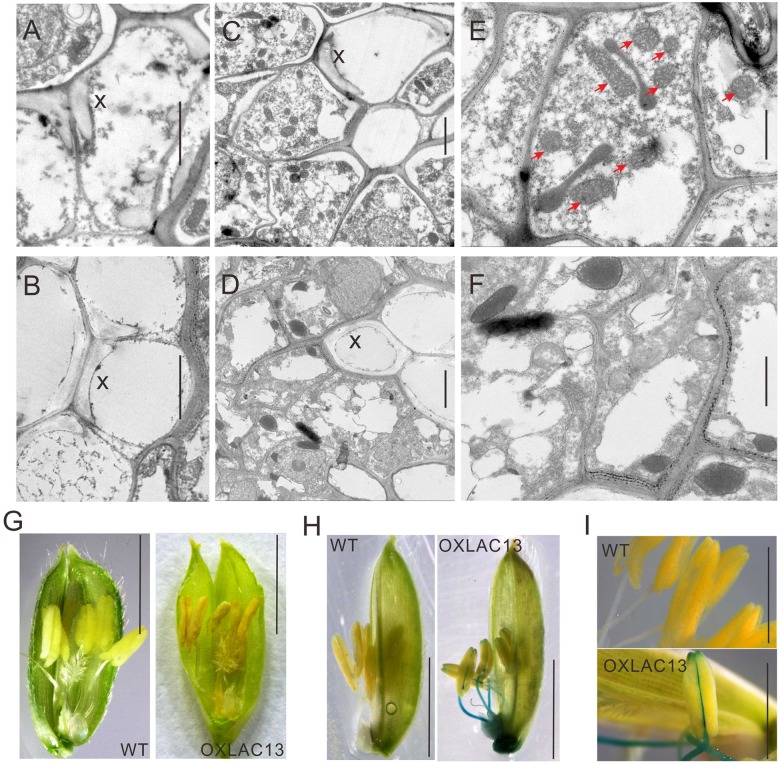
Transmission electron micrographs of vascular and ROS accumulation in WT and OXLAC13 anthers. **(A,B)** The secondary xylem of WT **(A)** and OXLAC13 **(B)** anther vascular. Scale bars, 2 μm. **(C,D)** The phloem of WT **(C)** and OXLAC13 **(D)** anther vascular. Scale bars, 2 μm. **(E,F)** Magnified image of the phloem of WT **(E)** and OXLAC13 **(F)**. The red arrows show the normal mitochondria in WT phloem (**E**). Scale bars, 1 μm. X, secondary xylem. **(G)** Flowers of WT and OXLAC13 plants at the mature stage, showing that the OXLAC13 flowers turn brown at this stage. Scale bars, 4 mm. **(H)** TMB staining of H_2_O_2_ production at the late anther development stage showing blue color. Scale bars, 4 mm. **(I)** A higher-magnification image of the anthers at the late anther development stage after TMB staining. Scale bars, 2 mm.

The observations above showed that the OXLAC13 plants might have defects in carbohydrate transport to anthers, and anther vascular tissues is responsible for transporting nutrients to anthers. We also found that the filaments in about 43.9% of the OXLAC13 anthers also failed to elongate (**Figure 2B**). Thus we speculated that the OXLAC13 plants might have abnormal anther vascular tissue and filaments, which restrained carbohydrate transport to anthers and failed to lift anthers to the top of the OXLAC13 spikelets. Laccases regulate lignin synthesis and the lignification of xylem in vascular bundles (Berthet et al., 2011; Lu et al., 2013; Zhao et al., 2013; Wang et al., 2014); moreover, vascular tissues transport carbohydrates. Therefore, we first used transmission electron microscopy to examine the vascular bundles in filaments and connectives of anthers. We observed only a slight increase of lignification in the secondary wall of vessels in the OXLAC13 plants compared with that of the WT plants (**Figures 3A,B** and Supplementary Figure 3E), and the vascular bundle showed no apparently abnormalities in morphology (Supplementary Figure 3E), which suggested that the sterility in the OXLAC13 plants might not be caused by the *OsLAC13* functions that associated with lignification. This phenomenon is similar to that observed in *Arabidopsis*, in which loss of function of only one *LAC* gene fails to induce an apparent phenotype in vascular bundles (Berthet et al., 2011).

As sugars are transported by phloem, we then observed the ultrastructure of the phloem cells in the WT and the OXLAC13 plants. Interestingly, we found that the WT phloem cells have lots of mitochondria with clear mitochondrial cristae (**Figures 3C,E**), but in the OXLAC13 phloem, the mitochondria are anomalous and seem to undergo degradation. We observed very few organelle with mitochondrial cristae in the OXLAC13 phloem cells during starch deposition in pollen during late anther development (**Figures 3D,F**), suggesting that *OsLAC13* could affect the number and integrity of mitochondria in the stamen vascular cells.

We also noticed that 48% of the OXLAC13 anthers started to turn brown before anthesis (**Figure 3G**). Anomalous organelles and brown anthers might indicate oxidative stress caused by higher levels of ROS, which cause oxidative damage to cellular structures and molecules (Gechev et al., 2006). Superoxide anion, hydrogen peroxide, and hydroxyl radicals are major ROS in plants (Hu et al., 2011). Given that hydroxyl radicals are unstable and difficult to detect directly in biological samples (Hu et al., 2011), we detected superoxide anion and hydrogen peroxide in the WT and the OXLAC13 anthers during development. Measurement of superoxide anion with nitroblue tetrazolium (NBT) showed no obvious differences in superoxide anion between the WT and the OXLAC13 stamens (Supplementary Figure 4A). We then used 3, 5, 3, 5′-tetramethylbenzidine (TMB) to analyze the cellular localization of hydrogen peroxide in anthers. Intriguingly, although we observed no obvious difference between the anthers of the WT and the OXLAC13 plants during early anther development (Supplementary Figure 4A), we did observe apparent differences during late anther development. Hydrogen peroxide started to accumulate in the filaments and the connectives of the OXLAC13 anthers in late anther development, when no hydrogen peroxide was detected in the WT anthers (**Figures 3H,I**).

To verify whether *OsLAC13* induces H_2_O_2_ accumulation in cells, we performed *in vitro* experiments in both rice cells and mammalian cell, respectively. *OsLAC13* was transfected into the mammalian expression system HEK-293T or rice protoplast cells. As expected, *OsLAC13* could induce apparent H_2_O_2_ accumulation in both the rice protoplast cells and the HEK-293T cells after 24 h or 48 h transfection, respectively (Supplementary Figure 5). These results showed that H_2_O_2_ accumulation was directly or indirectly driven by *OsLAC13* but not by biotic and abiotic stresses during development in the OXLAC13 plants.

H_2_O_2_ has been reported to affect mitochondrial functions that lipid peroxidation mediated by the interaction between ROS and membrane lipids can affect mitochondrial membrane integrity (Keunen et al., 2011). Mitochondria are required for plant development and they supply cellular energy by respiration for various biological processes, including sugar transport in phloem, and the respiratory rate in the phloem cells is much higher than in most of other tissues (Kuhn and Grof, 2010; Eom et al., 2012). In anthers, mitochondria also supply essential energy for elongation of filaments during flowering. These results suggest that *OsLAC13* is likely to promote hydrogen peroxide production in filaments and the anther connectives, and in turn affects the integrity of the mitochondria in the stamen vascular cells and might be account for pollen development. We further analyzed three OXLAC13 lines with different *OsLAC13* expression level, and found that a higher *OsLAC13* expression level induced a slightly higher H_2_O_2_ production, and a lower seed setting rate (Supplementary Figures 4C–E). This result suggested that the induction of H_2_O_2_ by *OsLAC13* in anthers might subsequently affected seed setting rate.

### Hydrogen Peroxide Dynamics and Oxidative Stress Responses Are Affected in the Transgenic Plants that Over-Expressing of OsmiR397, the Mediator of *OsLAC13*

**FIGURE 4:**
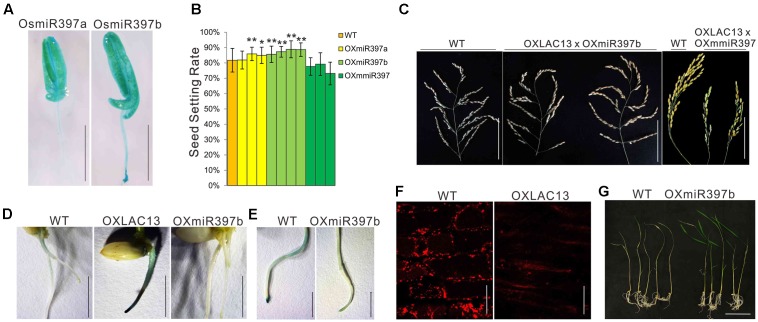
*OsLAC13* and its mediator OsmiR397 regulate hydrogen peroxide dynamics during root growth and stress response. **(A)** Expression patterns of OsmiR397a and OsmiR397b by GUS staining (Blue). Scale bars, 2 mm. **(B)** The seed setting rates of WT, OXmiR397a, OXmiR397b, and OxmmiR397 plants. Values are the means ± s. d. (*n* = 40 plants). Significant differences were identified at the 5% (^∗^) and 1% (^∗∗^) probability levels using Student’s *t*-test. **(C)** The panicles of OXLAC13 plants crossed with OXmiR397b and OxmmiR397 plants, respectively. Scale bars, 10 cm. **(D)** TMB staining showed abnormal H_2_O_2_ deposited at the root tips of OXLAC13 seedling, whereas slight H_2_O_2_ deposited in the WT roots and no H_2_O_2_ was observed in the OXmiR397 roots. Scale bars, 5 mm. **(E)** TMB staining of the WT and the OXmiR397 roots after 24 h MV treatment. Scale bars, 2 mm. **(F)** The mitochondria in WT and OXLAC13 root tips were stained by Mito Tracker red. No evident mitochondrion was observed in the OXLAC13 root tip. Scale bars, 20 μm. **(G)** The WT and OXmiR397b seedlings after 1 days MV treatment and 3 days recovering. Scale bars, 5 cm.

*OsLAC13* negatively regulates pollen development by promoting H_2_O_2_ production, that it might be suppressed by other molecule to ensure normal anther development. *OsLAC13* has been reported to be cleaved by OsmiR397 in rice. We first analyzed the expression patterns of OsmiR397 in anthers, and found it was highly expressed in filaments, anther connectives and pollen sacs (**Figure 4A**). The biological role of OsmiR397 in anthers might be suppressing *OsLAC13* expression and maintaining modest H_2_O_2_ level and seed setting rate. Consistent with the *OsLAC13* knockout plants, the seed setting rate increased in the OsmiR397 over-expressing plants (OXmiR397), but not in the transgenic plants that over-expressing mutated OsmiR397 gene (OXmmR397) (**Figure 4B** and Supplementary Figures 1B,C), which contains several mismatches to the *OsLAC13* binding site and couldn’t cleave the mRNA of *OsLAC13*. The hybrids of the OXLAC13 plants and the OXmiR397 plants have similar seed setting rate with that of the WT plants, whereas the hybrids of the OXLAC13 plants and the OxmmiR397 plants have low seed setting rate (**Figure 4C**), implying that the cleavage of *OsLAC13* by OsmiR397 is essential for seed setting rate maintenance in rice plants.

We next ask if OsmiR397 mediated *OsLAC13* also regulates H_2_O_2_ producing and mitochondrial function in other tissues. We found that, compared with the wild-type plants, the rice plants with ectopic expression of *OsLAC13* showed lethal phenotypes, and the lethal plants had very few or no root (Supplementary Figure 4B). It has been evidenced that excess H_2_O_2_ is harmful to organisms, and we then analyze the H_2_O_2_ contents and mitochondrial functions in the OXLAC13 roots. We detected the cellular localization of H_2_O_2_ by TMB staining after seed germination. The OXLAC13 roots were strongly stained 5 days after germination especially at the root tips, and the WT roots were slightly stained at the zone of maturation and no staining could be observed at the root tips, whereas the OXmiR397 plants showed no staining throughout the roots (**Figure 4D**), indicating that *OsLAC13* started to promote H_2_O_2_ production from the beginning of the plant development, and OsmiR397 reduced H_2_O_2_ storage. To monitor the effect of the elevated H_2_O_2_ contents in the OXLAC13 root tips on mitochondrial functions, we then detected active mitochondria in the root tip cells in which active mitochondria can be effectively stained using Mito Tracker red (Molecular Probes) as a marker. Consistently, the active mitochondria were not evident in the root tip of the poorly grown OXLAC13 seedling, while the active mitochondria distributed homogeneously in the WT root tip (**Figure 4F**).

It is known that oxidative stresses could induce H_2_O_2_, thus to further verify the effects of OsmiR397 on H_2_O_2_ dynamic, we then used methyl viologen (MV) to induce oxidative stress to the roots of the WT and the OXmiR397 plants, and detected the contents of H_2_O_2_. After 24 h 20 μM MV treatment, the root tips of the WT plants showed strong TMB staining, but the OXmiR397 root tips only showed slightly staining (**Figure 4E**). Consistently, after 3 days recovering, most of the OXmiR397 seedlings survived while most of the WT seedlings died (**Figure 4G**). It has been reported that miR397 is induced by different stresses in various species (Sunkar and Zhu, 2004; Wang et al., 2013; Maeda et al., 2016). Thus OsmiR397 regulated *OsLAC13* abundance might play a role in stress response by suppressing H_2_O_2_ production in roots. These data showed that OsmiR397 directs *OsLAC13* down-regulation is involved in affecting H_2_O_2_ dynamics during development and stress responses.

In conclusion, our results showed that *OsLAC13* regulates seed setting rate and plant growth in rice through promoting the production of hydrogen peroxide, and induce mitochondrial disruption. This process is under the regulation of OsmiR397. In stamen, *OsLAC13* might suppress sugar transport to the pollen grains and prevent the elongation of the filaments, which then lead to decreased seed setting rate.

## Discussion

### A Novel Function of Laccase in Plant Seed Setting Rate Regulation

Laccases belong to a large group of enzymes termed the blue copper proteins, including ascorbic acid oxidase and plastocyanin. Plant laccases are well-known to be involved in lignin synthesis (Turlapati et al., 2011; Cesarino et al., 2013; Lu et al., 2013; Zhao et al., 2013; Awasthi et al., 2015). Recent studies also showed *laccase* genes regulate seed yield in rice and in *Arabidopsis* (Zhang et al., 2013; Wang et al., 2014). In this study, we found a new function of Os*LAC13* in regulating H_2_O_2_ dynamics and rice seed setting rate. Knock-out or knock-down of *OsLAC13* increased seed setting rate, while overexpression of *OsLAC13* lead to abnormal male reproductive organogenesis by promoting H_2_O_2_ accumulation in filaments and connectives of anthers and affecting the integrity of mitochondria in the vascular tissues. We also showed the regulatory role of *OsLAC13* is under the control of OsmiR397 (Zhang et al., 2013). High expression level of OsmiR397 increases seed setting rate and restores the semi-sterility of the rice plants caused by high abundance of *OsLAC13* mRNA.

We have reported that *OsLAC13* suppressed brassinosteroid (BR) signaling and in turn affected grain yield in rice (Zhang et al., 2013). BRs are also essential for male fertility in plants. Some studies showed that BRs are involved in pollen tube and filament elongation by regulating related genes (Szekeres et al., 1996; Bouquin et al., 2001; Li et al., 2001; Kim et al., 2005; Ye et al., 2010), although the detailed molecular mechanisms have not been identified. In this study, we identified that *OsLAC13* regulates seed setting rate and male reproductive organogenesis through H_2_O_2_ pathway. Whether BR signaling is also involved in the *OsLAC13*-mediated process has not been demonstrated and more efforts are necessary to further investigate the functions of this gene. These findings indicated that laccases in higher plants are much more complicated than previously estimated.

### ROS in Male Reproductive Organogenesis

Most ROS molecules form as toxic by-products of aerobic metabolism in plants subjected to abiotic stresses (Bailey-Serres and Mittler, 2006). In recent years, studies have discovered the important roles of ROS in regulating plant development (Miller et al., 2008). ROS, particularly H_2_O_2_, serve as signaling molecules in diverse processes (Quan et al., 2008), such as cell expansion (Suzuki et al., 1999), apical dominance (Semighini and Harris, 2008), senescence (Cui et al., 2013; Allu et al., 2014), and flower development (Zafra et al., 2010; Hu et al., 2011; Kaya et al., 2014; Schippers et al., 2016), as well as stress responses (Xia et al., 2010; Baxter et al., 2014; Considine et al., 2015).

Several recent studies have reported the close connection between ROS and male reproductive organogenesis in plants. For example, Hu et al. (2011) studied the roles of the transcriptional regulator *MADS3* in male sterility. Mutation of rice *MADS3* affects its regulation of MT-1-4b, which has superoxide anion and hydroxyl radical-scavenging activity; the resulting increased level of superoxide anion causes decreased pollen fertility (Hu et al., 2011). Wu et al. (2010) reported the roles of H_2_O_2_ in pollen tube growth, showing that spermidine-derived H_2_O_2_ signals Ca^2++^ influx and thereby regulates pollen tube growth. ROS are also critical for tapetal programmed cell death and pollen development, and excessive accumulation of ROS also occurs in the anthers of cytoplasmic male sterile rice (Jiang et al., 2007; Luo et al., 2013; Xie et al., 2014; Li et al., 2015). In general, the appropriate amount of ROS in the tapetum is crucial for generating fertile anthers in plants, but abnormal production of ROS can harm the male reproductive organ (Hu et al., 2011; Xie et al., 2014). In this study, we reported that overexpression of *OsLAC13* could induce the accumulation of H_2_O_2_ in the filaments and connectives of anthers, which then affected mitochondrial integrity in the vascular tissue cells. Our findings showed that ROS accumulation in filaments also affects male reproductive organogenesis in plants, and in turn affects grain setting rate.

### The Possible Mechanism of *OsLAC13* in Hydrogen Peroxide Production and Deposition in Plant

In plant laccases are proposed to function in the formation of lignin by promoting the oxidative coupling of monolignols, a family of naturally occurring phenols (Berthet et al., 2011; Lu et al., 2013; Zhao et al., 2013; Wang et al., 2014). The range of substrates which various laccases can attack is very wide, and substrates similar to a p-diphenol will be oxidized by laccases (Mayer and Staples, 2002). This characteristic makes the actions of laccases much complicated that up to now most functions of laccases in plants remain largely unknown.

Fungi laccases have been reported to catalyze reduction of molecular oxygen to H_2_O, but not H_2_O_2_ (Morozova et al., 2007). However, the catalytic activity of plant laccases is not well identified. It has been reported that laccases in different species have a fairly broad but distinctive substrate spectrum amongst the enzymes (Reiss et al., 2013). Thus, whether plant laccases could produce H_2_O_2_, and which substrate might be responsible for H_2_O_2_ production have not been reported yet.

In the study, we showed that H_2_O_2_ could be a product of a rice laccase catalysis, showing the differences between plant and fungi laccases. *In vitro* experiments showed that expressing *OsLAC13* in mammalian cells could induce H_2_O_2_ production independently. This result indicated that OsLAC13 regulates H_2_O_2_ with no need of other plant specific protein. It has also been reported that, in plant, peroxidase uses ascorbate as the reductant to remove H_2_O_2_ (Noctor et al., 2000). Importantly, *OsLAC13* and L-ascorbate oxidase belong to the blue oxidase family. Moreover, *OsLAC13* has over 85% similarity with the L-ascorbate oxidase in *Zea mays*, implying that plant laccase, at least *OsLAC13* could oxdize the reductant of peroxidase to restrain H_2_O_2_ removal. We have compared the ascorbate contents in the OXLAC13, the OXmiR397a/b and the WT plants. Interestingly, the ascorbate content was lower in the OXLAC13 plants and was higher in the OxmiR397a/b plants compared with that of the WT plants, negatively associated with the content of H_2_O_2_ (Supplementary Figure 3D). These data suggested that plant laccase might induce hydrogen peroxide deposition although further studies are necessary before the conclusion was made especially the enzyme substrates need to be declared.

### Importance of Filaments and Connectives of Anthers in Regulating Crop Seed Setting Rate

Crop domestication is essential for food supply, and increasing seed setting rate is critical for crop domestication. Except environmental factors, reproductive organogenesis and pollination are determinants of seed setting rate. Male reproductive organogenesis include both the early phase of stamen formation and morphogenesis and the late phase of pollen grain maturation, stamen filament elongation, and anther dehiscence (Cardarelli and Cecchetti, 2014). Most studies of the male reproductive organogenesis focused on meiosis during pollen formation, the roles of the tapetum in pollen maturation, and the process of pollen tube elongation, but studies on filaments and connectives of anthers remain limited. Filaments and connectives function as conduits for water and nutrients and as a support that elongates to allow pollen deposition on the receptive stigma. The importance of filaments and connectives of anthers in male reproductive organogenesis and seed setting rate regulation has not been specifically studied yet.

Pollen development depends on energy supply. Mitochondria produce ATP via respiration and are essential for cellular energy production (Chen and Liu, 2014; Horn et al., 2014). The biogenesis of the sporophytic and gametophytic cells of plant anthers is thought to demand more cellular energy than other organs (Chen and Liu, 2014) and thus anthers have a lot of mitochondria. Some cytoplasmic male sterility genes cause mitochondrial dysfunction where the mitochondria fail to provide enough ATP for male development; this dysfunction then induces sterility (Ling et al., 1979). Carbohydrate transport in the phloem also needs energy, which is generated by mitochondrial respiration (Kuhn and Grof, 2010; Eom et al., 2012), and phloem tissues usually have a large number of mitochondria to support nutrient transport. The filaments and connectives of anthers have clear importance for supplying nutrients during anther development. In this study, we showed that the abnormal accumulation of ROS in the filaments and connections of anthers could injure the integrity of mitochondria, and this might decrease the energy supply available for carbohydrate transport and filament elongation. We indeed observed a blockage of carbohydrate transport from shells to anthers and a failure of filament elongation in some OXLAC13 plants. Thus it could be proposed that proper energy supply by mitochondria in filaments and connectives of anthers aids male reproductive organogenesis and improves seed setting rate in plants.

## Author Contributions

YY, Q-FL, and J-PZ carried out mutant screening and validation experiments. FZ performed ultrathin section experiments and Y-FZ and Y-ZF carried out histochemical assays. Y-QC conceived of the study, and participated in its design and coordination and helped to draft the manuscript. Y-CZ designed and carried out the functional analysis and drafted the manuscript. All authors read and approved the final manuscript.

## Conflict of Interest Statement

The authors declare that the research was conducted in the absence of any commercial or financial relationships that could be construed as a potential conflict of interest.
