# Supplementary material for: Laccase-13 Regulates Seed Setting Rate by Affecting Hydrogen Peroxide Dynamics and Mitochondrial Integrity in Rice
Source: Front Plant Sci. 2017 Jul 26;8:1324. doi: 10.3389/fpls.2017.01324 (PMC5526905; doi:10.3389/fpls.2017.01324)
Supplement: Supplementary file 1 [file Image_1.PDF]

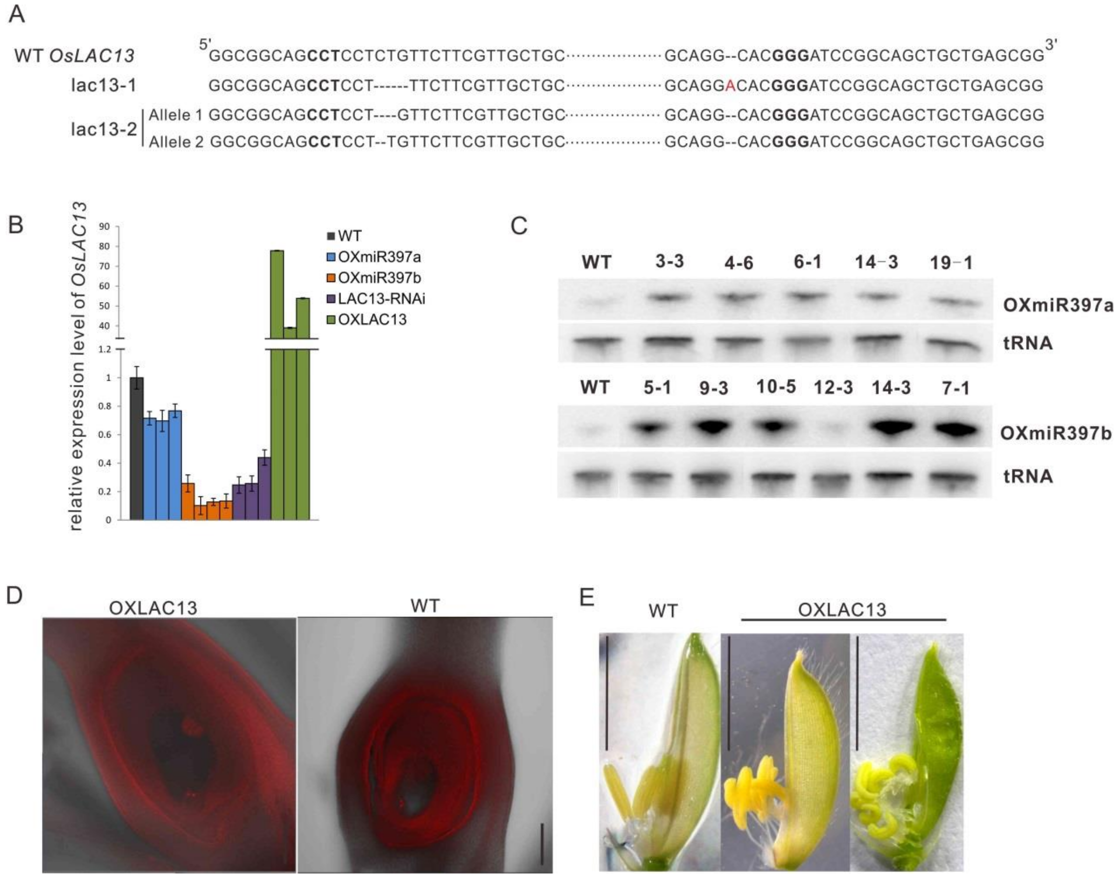

Supplementary Figure 1. Transgenic plants used in this study and their phenotypes. (**A**) The genotypes of the *OsLAC*13 knock-out mutants. (**B**) The expression level of *OsLAC13* in different transgenic lines. Values are the means ±s.d. (**C**) The expression level of OsmiR397a and OsmiR397b in different transgenic lines by northern blot. (**D**) The pistils of WT and OXLAC13 plant. (**E**) The spikelets of the OXLAC13 plants.
