# Supplementary material for: Laccase-13 Regulates Seed Setting Rate by Affecting Hydrogen Peroxide Dynamics and Mitochondrial Integrity in Rice
Source: Front Plant Sci. 2017 Jul 26;8:1324. doi: 10.3389/fpls.2017.01324 (PMC5526905; doi:10.3389/fpls.2017.01324)
Supplement: Supplementary file 2 [file Image_2.PDF]

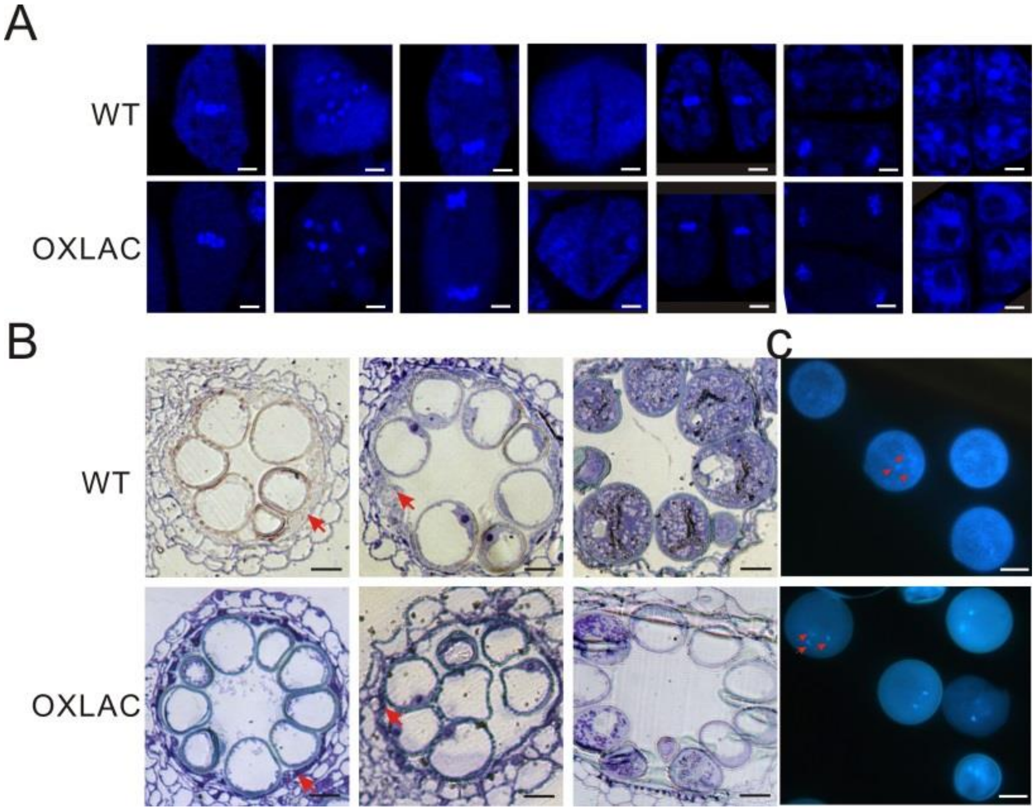

Supplementary Figure 2. The early development of WT and OXLAC13 pollen. (**A**) Chromosome dynamics in the WT and OXLAC13 pollen mother cells. Chromosomes are stained with 1 μg/ml DAPI. Scale bars, 5 μm. (**B**) Transverse sections of WT and OXLAC13 anthers after meiosis. Scale bars, 15 μm. (**C**) The mature pollen grains of WT and OXLAC13 plants stained with 1 μg/ml DAPI. The red arrows indicate the nucleus of the mature pollen. Scale bars, 15 μm.
