# Supplementary material for: Laccase-13 Regulates Seed Setting Rate by Affecting Hydrogen Peroxide Dynamics and Mitochondrial Integrity in Rice
Source: Front Plant Sci. 2017 Jul 26;8:1324. doi: 10.3389/fpls.2017.01324 (PMC5526905; doi:10.3389/fpls.2017.01324)
Supplement: Supplementary file 3 [file Image_3.PDF]

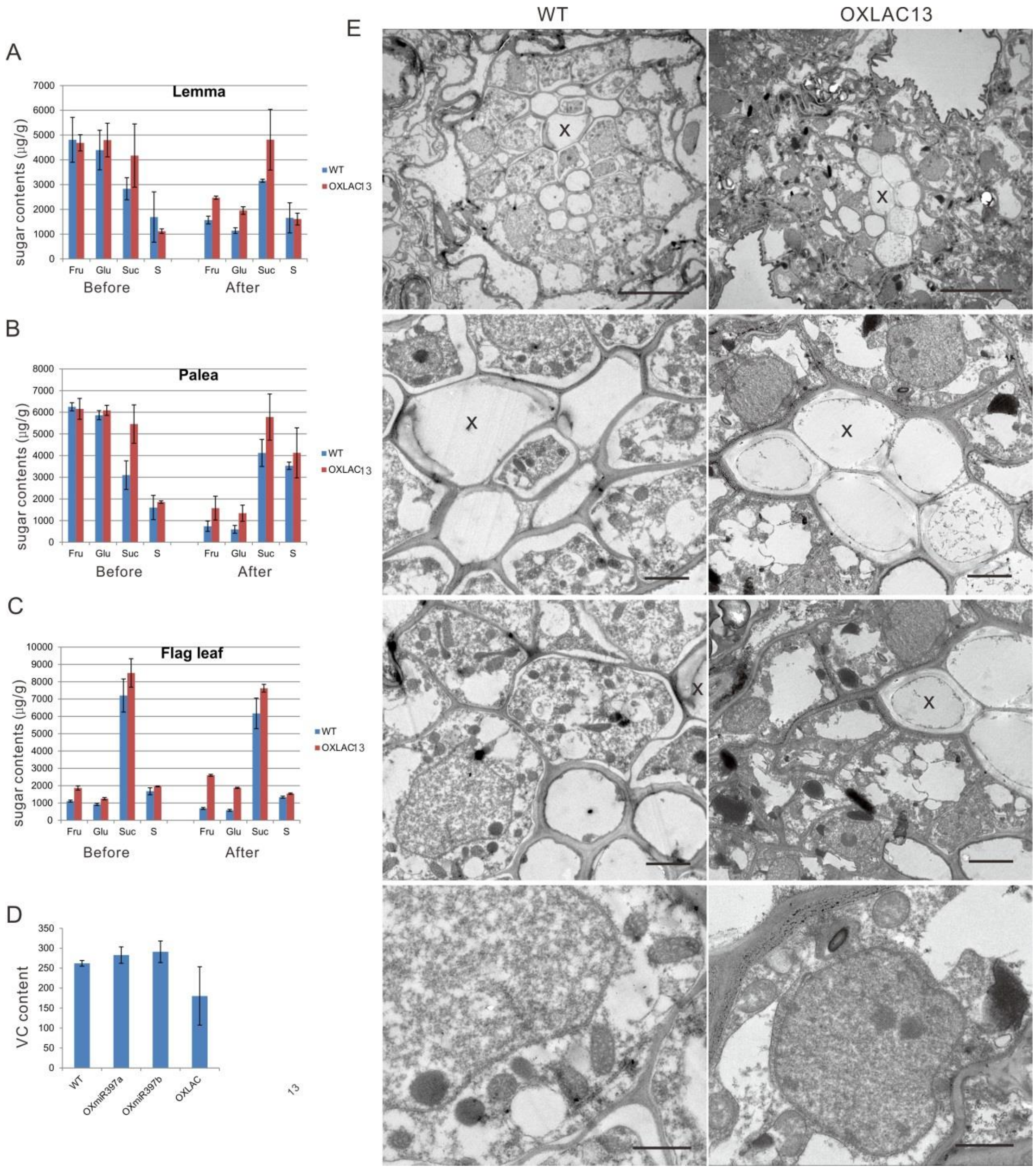

Supplementary Figure 3. Sugars, starch and ascorbate levels in the WT and OXLAC13 plants. (**A-C**) Sugar and starch levels before and after starch deposition in pollen grains in flag leaf, lemma and palea. Values are the means ± s. d. (n = 3 replicates) with units of µg/g fresh weight (FW). Fru, fructose; Glu, glucose; Suc, sucrose; S, starch. (**D**) The ascorbate contents of WT, OXLAC13 and OxmiR397a/b seedlings. Values are the means ± s. d. (n = 3 replicates) with units of ppm/g fresh weight (FW). (**E**) Transmission electron micrographs of vascular in WT and OXLAC13 anthers. Scal bars are 10uM, 2uM, 2uM and 1uM respectively, from the top panels to the bottom panels. X, secondary xylem.
