# Supplementary material for: Laccase-13 Regulates Seed Setting Rate by Affecting Hydrogen Peroxide Dynamics and Mitochondrial Integrity in Rice
Source: Front Plant Sci. 2017 Jul 26;8:1324. doi: 10.3389/fpls.2017.01324 (PMC5526905; doi:10.3389/fpls.2017.01324)
Supplement: Supplementary file 4 [file Image_4.PDF]

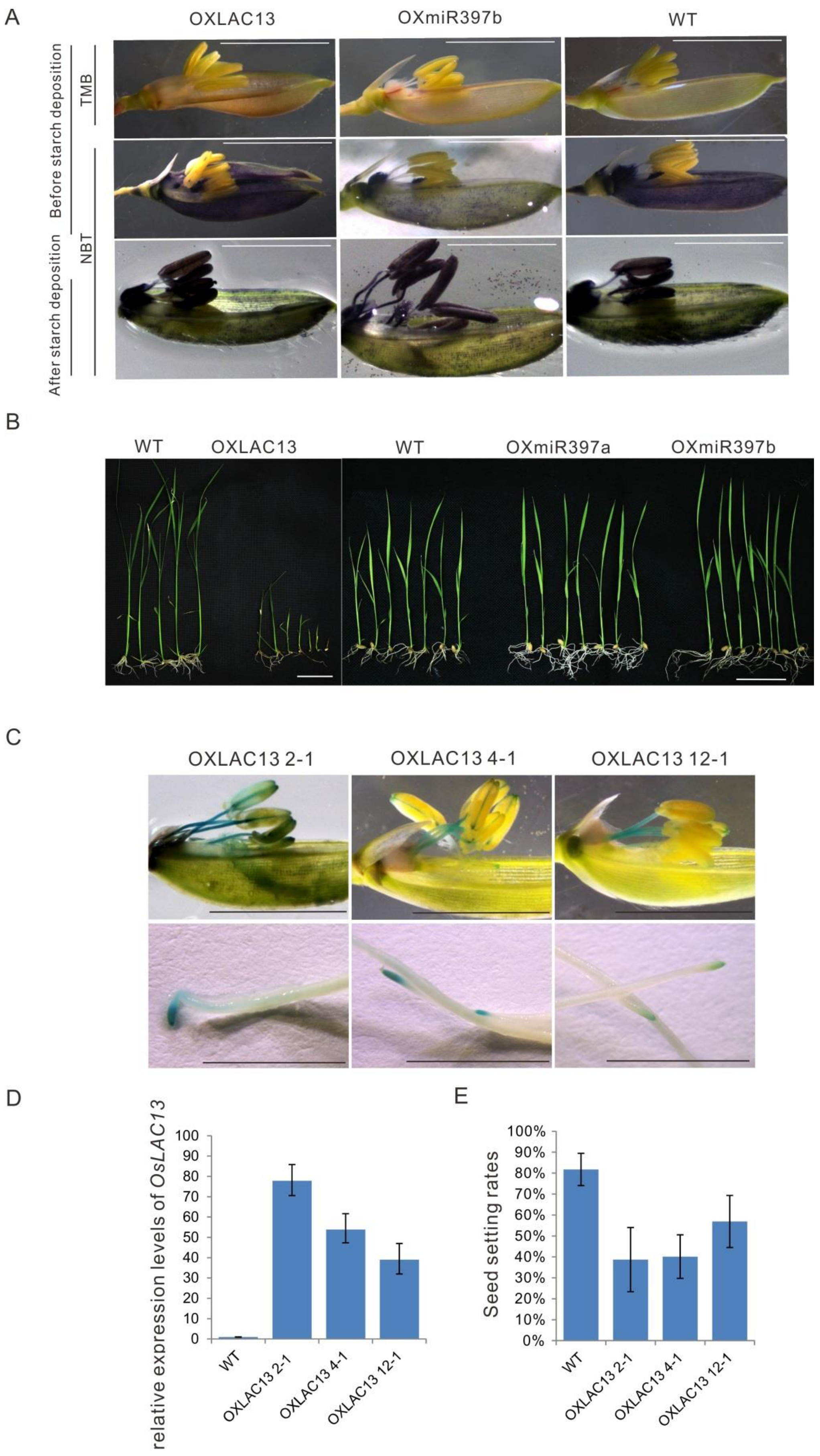
A
OXLAC13
OXmiR397b
WT
Before starch deposition
After starch deposition
TMB
NBT
B
WT
OXLAC13
WT
OXmiR397a
OXmiR397b
C
OXLAC13 2-1
OXLAC13 4-1
OXLAC13 12-1
D
relative expression levels of OsLAC13
0
10
20
30
40
50
60
70
80
90
100
WT
OXLAC13 2-1
OXLAC13 4-1
OXLAC13 12-1
E
Seed setting rates
0%
10%
20%
30%
40%
50%
60%
70%
80%
90%
100%
WT
OXLAC13 2-1
OXLAC13 4-1
OXLAC13 12-1

Supplementary Figure 4. Analysis of H_2O_2 and $O_2^{\bullet -}$ (dark blue) levels in early and late anther development in WT and OXLAC13 anthers, and the phenotypes of of the WT, OXLAC13 and OXmiR397 seedlings. (**A**) Analysis of H_2O_2 levels by TMB staining in early anther development and analysis of $O_2^{\bullet -}$ levels by NBT staining in early and late anther development showing dark blue color in WT and OXLAC13 anthers. Scale bars, 4 mm. (**B**) The seedlings of WT, OXmiR397 and OXLAC13 plants. Scale bars, 5 cm. (**C**) H_2O_2 levels in anthers and roots in different OXLAC13 transgenic lines. Scale bars are 4mm for anthers and 5mm for roots. (**D**) The expression level of OsLAC13 in different OXLAC13 transgenic lines. (**E**) Seed setting rates of different OXLAC13 transgenic lines.
