# Supplementary material for: Laccase-13 Regulates Seed Setting Rate by Affecting Hydrogen Peroxide Dynamics and Mitochondrial Integrity in Rice
Source: Front Plant Sci. 2017 Jul 26;8:1324. doi: 10.3389/fpls.2017.01324 (PMC5526905; doi:10.3389/fpls.2017.01324)
Supplement: Supplementary file 5 [file Image_5.PDF]

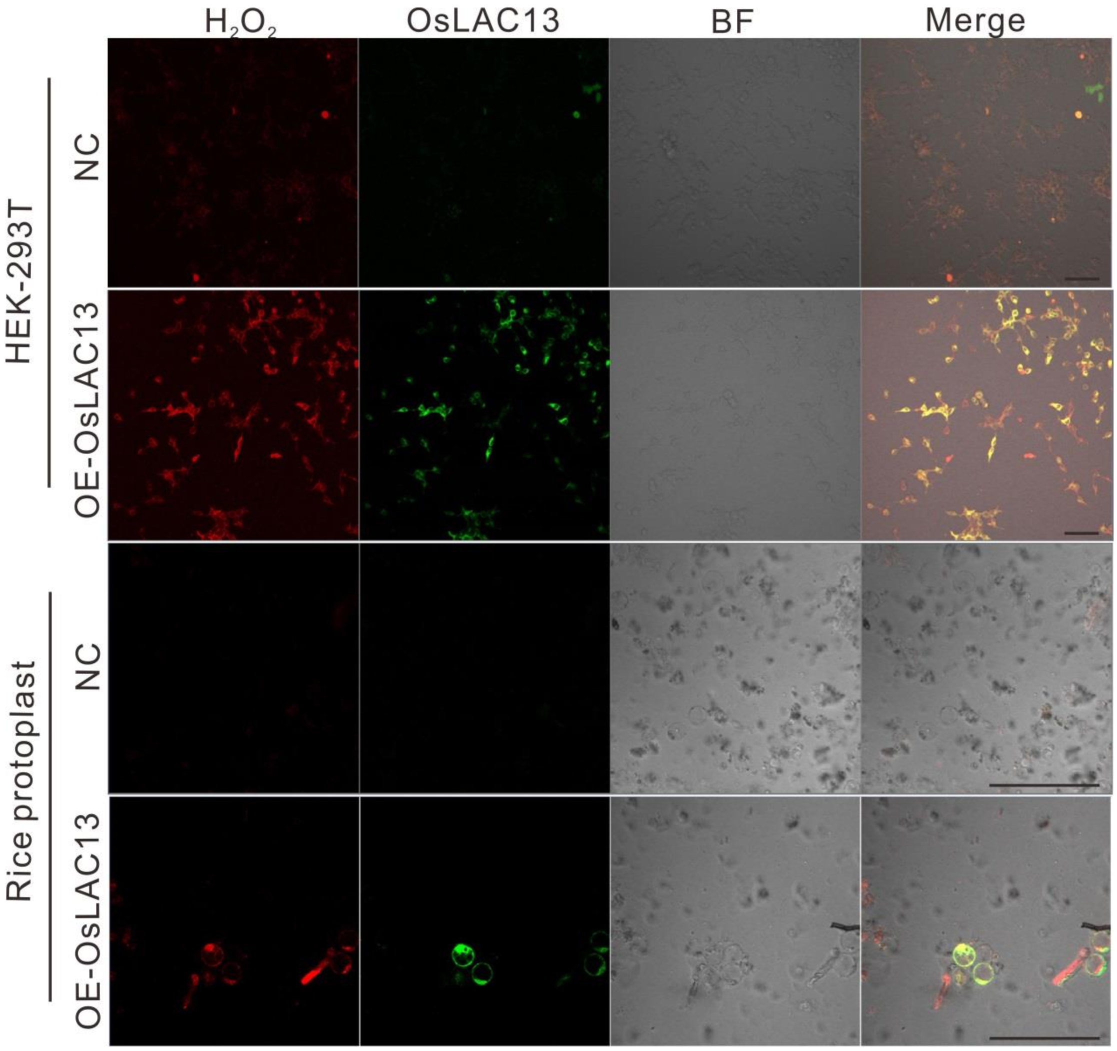

Supplementary Figure 5. *OsLAC13* induces H_2O_2 accmulation in vitro. OsLAC13-eGFP (Green) was over-expressed in HEK-293T cell line and rice protoplast respectively. H_2O_2 was indicated by Orp1-roGFP (Premo^{TM} Cellular hydrogen peroxide H_2O_2 Sensor, Molecular probes) (Red). Scale bar, 100 μM.
